# Antifungal effects of selected menthol and eugenol in vapors on green coffee beans during long-term storage

**DOI:** 10.1016/j.heliyon.2023.e18138

**Published:** 2023-07-10

**Authors:** Yamina Ben Miri, Ahmed Nouasri, Amina Benabdallah, Abderrahim Benslama, Zeynep Tacer-Caba, Affaf Laassami, Djamel Djenane, Jesus Simal-Gandara

**Affiliations:** a Department of Biochemistry and Microbiology, Mohamed Boudiaf University, BP 166 M'sila 28000, M'sila, Algeria; b Food Quality and Safety Research Laboratory, Department of Food Sciences. Mouloud Mammeri University; BP, 17. 15000, Tizi-Ouzou, Algeria; c Laboratory of Bioactive Products and Biomass Valorization Research. ENS Kouba, BP92, Kouba, Algiers, Algeria; d Laboratory on Biodiversity and Ecosystem Pollution, Faculty of Life and Nature Sciences. University Chadli Bendjedid, El-Tarf, 36000, Algeria; e Department of Molecular Biology and Genetics, Bahcesehir University, Besiktas, Istanbul, Turkey; f Microbial Systems Biology Laboratory (LBSM); ENS Kouba, BP92, Kouba, Algiers, Algeria; g Universidade de Vigo, Nutrition and Bromatology Group, Analytical Chemistry and Food Science Department, E32004 Ourense, Spain

**Keywords:** Green coffee beans, *A. parasiticus*, Menthol, Eugenol, Fumigation, Antifungal activity

## Abstract

Nowadays, coffee (Coffea Arabica L.) is among the most significant agricultural products of the world and drinking coffee has become one of the most popular habits in the world. The main contamination of stored coffee beans is related with the mycotoxin produced by the toxigenic fungi belonging the genus *Aspergillus*. Fungal infection followed by mycotoxin biosynthesis in coffee results in notable financial losses. subsequent mycotoxin biosynthesis in coffee leads to major economic losses. Complications ranging from mild to severe can be caused by the mycotoxins produced by this genus. The aim of this investigation was to determine the effect of menthol and eugenol on *Aspergillus parasiticus* (CBS 100926^T^) growth, spore germination, and their potential use as green coffee beans preservative during long-term storage (12 months). The minimum inhibitory concentrations (MICs) values of the menthol and eugenol were recorded to completely inhibit the growth of *A. parasiticus* in 400 μg/ml and 300 μg/ml, respectively. Both reduced spore germination by 9.33% and 5.66% at 300 μg/ml and 200 μg/ml, respectively. They showed efficacy in fumigated green coffee beans sample during the storage for up to 12 months providing an increase in the protection level of 62.5% for menthol and 73.21% for eugenol against the *A. parasiticus* contamination. This suggests that menthol and eugenol could be used as good alternatives for decreasing the deteriorations due to the fungal infections in green coffee beans during long-term storage.

## Highlights

• Antifungal activity of menthol and eugenol against the toxigenic strain of *Aspergillus parasiticus* was assessed.
• Menthol and eugenol decreased fungal growth of *A. parasiticus* at lower concentrations.
• Menthol and eugenol showed above 50% protection of green coffee beans from *A. parasiticus* contamination.
• Menthol and eugenol could be used as good alternatives for decreasing the deteriorations due to the fungal infections in green coffee beans during long-term storage.

## 1 Introduction

Coffee is among the most significant agricultural products of the world and coffee beverage is still considered enjoyable and valuable worldwide. The improvement and preservation of coffee quality are of critical importance, as the amount of sales mainly depends on the coffee quality.

Coffee is one of the most popular products in Algeria. Because all of the coffee is imported, they may become contaminated with toxigenic fungi during processing and storage. The prolonged storage of green coffee causes distinctive quality losses, those are mainly characterized by the cup's typical “flattening and slackening” [1]. These changes apparently are related to a reduction in the aroma potential in the green bean. Green coffee beans are prone to contamination over different processing phases [2,3]. Among all other spoilage agents, filamentous fungi represent the greatest health risk, with their potential to use harmful mycotoxins. In the absence of good practices, studies have shown that mycotoxins are present in a large percentage in coffee beans during storage, and even in the final drink [4]. Environmental conditions such as humidity and high temperatures promote the growth of fungi in stored coffee beans. As a consequence, good hygiene standards and manufacturing practices are strongly advised to decrease contamination risk in processed coffee [5]. The big concern in coffee production currently is climate change [6,7].

The genus *Aspergillus* contains the majority of the toxigenic fungi found in coffee [8] and the mycotoxins produced by this genus are characterized by their carcinogenicity, nephrotoxicity, immunotoxicity, hepatotoxicity, embryotoxicity, teratogenicity, and mutagenicity, in the literature [9]. Furthermore, the most important stage of strategy to protect the product from infection is the proper storage of green coffee beans after processing. Therefore, enhancement of the quality is only possible by preventing the adverse impacts of fungi contamination and their mycotoxins. Ochratoxin A (OTA) is the most extensively investigated mycotoxin growing in coffee [8,10,11]. However, other studies have also demonstrated the presence of aflatoxins (AFs) [9]. The synergistic effect of combining various mycotoxins is significant, and it occurs frequently in stored foodstuffs [6]. The International Agency for Research on Cancer (IARC) categorized OTA as a feasible carcinogen (group 2B) and AF as human carcinogens based on their carcinogenic effects (group 1). To protect the society from the effects of mycotoxin, developed countries have enacted mycotoxin regulations for both human food and animal feed.

The biological effect of essential oils (EOs) and their bioactive constituents is various. EOs have numerous uses in the food industry and agriculture [12]. Since EO are natural substances, they may also show some effects on fungal growth, sporulation, and even mycotoxin production [13]. As a result, they are proposed as promising and appropriate solutions to the problem of fungi and their mycotoxins contamination not only in coffee, but also in other food products.

There is survey on the occurrence of mycotoxins in the most commonly consumed traditional foods in Algeria and dietary exposure risk assessment [14]. Different food samples of couscous, wheat, nuts, metlou, figs, and Rechta were collected from daily markets, hypermarkets, groceries store, bakeries, cafeterias. Out of 198 samples, 82 (41.4%) were tested positive for at least one mycotoxin. As a result, the study recommended that the AFs concentration limits of authorized in foodstuffs in Algeria must be updated on a regular basis.

This study aims to look into the efficacy of menthol and eugenol on *A. parasiticus* growth, spore germination, and their potential use as green coffee bean preservatives over a long period of storage.

## 2 Materials and methods

### 2.1 Chemicals, solvents and culture media

Dimethyl sulfoxide (DMSO) and media comstituents *vis*. Potato Dextrose Agar (PDA) medium (Potato, 200 g; Dextrose, 20 g; Agar, 15 g and distilled water 1000 ml) were purchased from Aldrich Sigma (France). Commercial menthol (purity 99%) and eugenol (purity 98%), were purchased from Sigma-Aldrich (France). The components were maintained in glass opaque flasks at 4 °C until use. Green coffee beans (Coffea Arabica L.) were obtained from the Rouiba market in Algiers, Algeria.

### 2.2 Microorganism

*A. parasiticus* (CBS 100926^T^) was provided from Microbial Systems Biology Laboratory (LBSM), Kouba, Algiers and cultured on PDA medium at 28 ± 2 °C for 7 days. Spore inoculum (washing the 7-days culture of *A. parasiticus* with 20 ml of 0.1% Tween 80 solution) was prepared. Total spores were counted (approx. 1 × 10^6^ spores/ml) using a hemocytometer slide (depth 0.2 mm, 1/400 mm^2^) under a light microscope (Motic: BA210, China) throughout the study.

### 2.3 Different menthol and eugenol concentrations and their effects on the inhibition of *A. parasiticus*

The effect of menthol and eugenol on the mycelial growth of *A. parasiticus* (CBS 100926^T^) was assessed using the method as described of José Velázquez-Nuñez et al. [15]. The obtained solution was mixed with the agar medium to produce concentrations ranging from 100 to 400 μg/ml PDA medium. After solidification, 10 μl of the fungal suspension (approx. 1 × 10^6^ spores/ml) were accumulated in the culture medium. The control contained no menthol, eugenol, and their combination. The whole was incubated at 28 ± 2 °C. Mycelium growth was tracked by daily diameter measurement along two straight lines perpendicular to the center. The effectiveness of treatments was assessed at day 7. To determine the percentage of inhibition (I%), the following equation (1) was used:

(1) I% = (D_control_ – D_Test_ /D_control_) × 100

where D_Test_: Diameter of the test growth area in mm; D_control_: Diameter of the contol growth area in mm.

The minimum inhibitory concentration (MIC) and minimum fungicidal concentration (MFC) were determined for *A. parasiticus* (CBS 100926^T^) [16]. First of all, changing concentrations (100–400 μg/ml) of menthol and eugenol were included into 10 ml PDB liquid medium in test tubes. Control samples contained only PDB medium (10 ml). The inoculated tubes (containing 10 μl of spore suspensions) were incubated at 28 ± 2 °C for 7 days. The MIC was determined and then 100 μl of suspensions from the tubes were grown on PDA plates for MFC measurements.

### 2.4 Different concentrations of menthol and eugenol and their effects on spore germination of *A. parasiticus* (CBS 100926^T^)

Spores were collected by rubbing the mycelial surface with 5 ml of sterile distilled water containing 0.1% (v/v) Tween-80 from 7 day cultures of *A. parasiticus* (CBS 100926T) that had previously been exposed to menthol and eugenol (100–400 g/ml). For the control, the same procedure was used. Fungal spore suspensions were inoculated into fresh PDA medium in depression slides for 24 h at 28 °C. The spore germination percentage was determined [17].

### 2.5 Antifungal effect of menthol and eugenol vapors of stored coffee beans inoculated with *A. parasiticus* (CBS 100926^T^)

The fumigant efficacy of menthol and eugenol against *A. parasiticus* (CBS 100926T) in green coffee beans was evaluated as previously described by Prakash et al. [16]. The green coffee beans were surface-sterilized with a 1% solution of sodium hypochlorite, rinsed 3 times with sterilized distilled water, and dried. The antifungal effectiveness of menthol and eugenol was estimated by storing 1 kg separately in glass containers for 12 months at 15 °C and 60% relative humidity. Three milliliter of conidial suspension of *A. parasiticus* were uniformly sprayed on the green coffee beans. Then, menthol and eugenol were applied on sterile cotton swabs attached to the inner surface of the container stoppers and impregnated at their MIC values related to the air container volume. The containers containing the inoculated green coffee beans and the two compounds were tightly closed and sealed with parafilm. An identical group without compounds was served as a control.

For the mycological analysis, the spread-plate method was used. Treated and control samples (10 g each) were mechanically homogenized for 15 min in 250 ml flasks containing 90 ml of sterile Tween water (0.1%). Serial decimal dilutions (up to 10^−3^) of 0.1 ml (each), was plated and uniformly distributed on freshly prepared PDA. Inoculated Petri dishes were incubated at 28 ± 2 °C for 5 days [18]. The percentage of protection provided by coffee beans was calculated using the number of isolates of *A. parasiticus* found in treated and control samples, using formula (2):

(2) $$\%P=\frac{Dc-Dt}{Dc}\times 100$$

%P = Protection percentage, Dc = Total number of *A. parasiticus* isolates from samples of control, Dt = Total number of *A. parasiticus* isolates from samples in treatment.

### 2.6 Statistical analysis

All data were expressed as mean ± standard deviations, and experiments carried out in triplicates. Analysis was conducted by one-way analysis of variance (ANOVA) and Tukey's post hoc test (*p* < 0.05) using STATISTICA software (Version 6).

## 3 Results and discussion

**Fig. 1:**
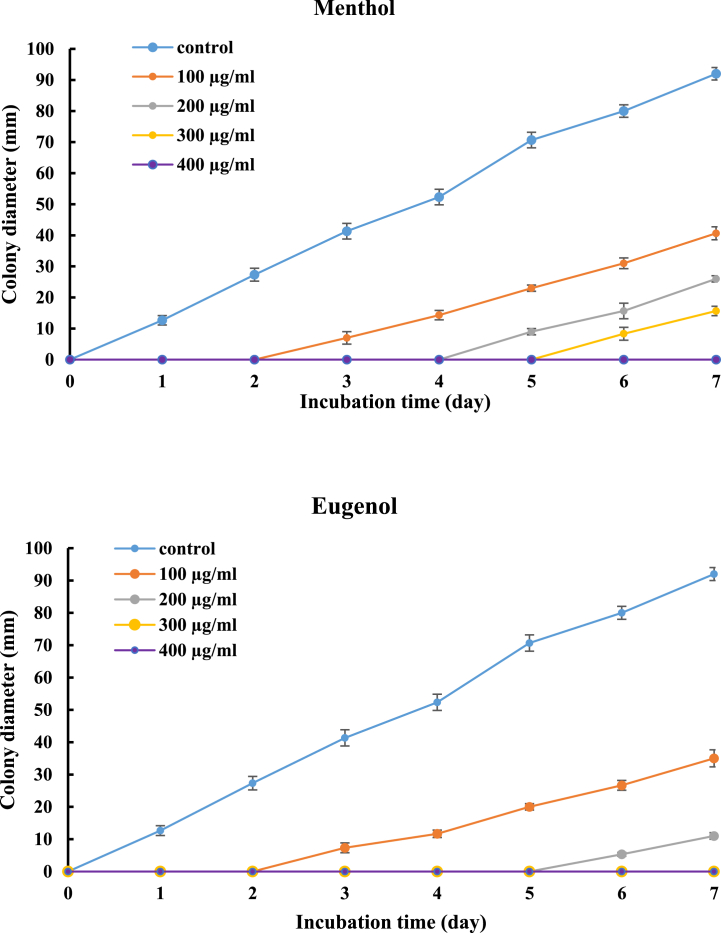
Effect of different concentrations of menthol and eugenol on the growth of *A. parasiticus* (CBS 100926^T^) during 7 days. Values are means (n = 3) ± SD.

**Fig. 2:**
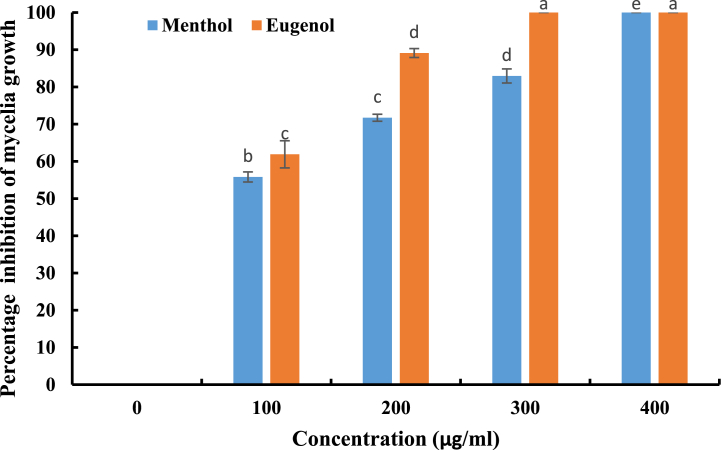
Percent inhibition of mycelial growth of *A. parasiticus* (CBS 100926^T^) after 7 days. Values are means (n = 3) ± SD.

Menthol and eugenol have been shown to inhibit the growth of *A. parasiticus* (CBS 100926T) during the incubation period (7 days) as depicted in Fig. 1. Mycelium growth was significantly (*p* < 0.05) reduced in proportion to concentrations for all treatments, indicating dose-dependent activity, although the growth gradually increased over time. Mycelial growth was delayed by five days for menthol and for eugenol at 300 μg/ml and 200 μg/ml, respectively. On the seventh day, the percent inhibition of mycelia growth was determined, as shown in Fig. 2. The findings revealed that the inhibition percentage of *A. parasiticus* (CBS 100926T) relative to the control, was in the range of 55.81–82.94% for menthol and 61.89–89.11% for eugenol (*p* < 0.05).

Major components of EOs *vis.* Thymol, eugenol, anethole, menthol, citral, pinenes, cinnamaldhydes, carvacrol, and carvone have been extensively studied concerning their antimicrobial spectrum against different microorganisms, including bacteria and fungi [19,20]. Thus, eugenol is a phenolic component that is mostly taken from buds and leaves of clove and from cinnamon. This component has been thoroughly studied for its antimicrobial properties against a broad range of microorganisms [21]. Also, Prakash et al. [22] found that eugenol, the main constituent of *Piper betle*, was more potent as a fungal growth inhibitor than the whole EO. According to Abbaszadeh et al. [23], eugenol inhibited the growth of *Cladosporium* sp. and *Aspergillus* sp. significantly. On other hand, Soković et al. [24] reported the effecacy of *Mentha piperita* EO and its constituents against various foodborne fungi. *Mentha* EO exhibited potent antifungal activity, but less than pure menthol. Mishra et al. [25] reported that *A. flavus* presented sensibility to menthol on all tested concentrations. The research by Shin et al. [26] showed that thymol and linalool were effective on the significant inhibition of mycelial growth of Botrytis cinerea. In addition, Dammak et al. [27] showed that 1,8-cineole inhibited the mycelial growth of *A. carbonarius*. Eugenol, D- and l-limonene, α-pinene, nerol against *A. niger*, *A. ochraceus* and *A. flavus* have been also studied for their antifungal activity, by Mihai and Popa [28].

Molecules with a hydroxyl groups (menthol) and a non-localized electron system (eugenol) in the phenolic ring structure exhibited significant antifungal activity [29]. Studies have displayed that the main antimicrobial mechanism involves increased the permeability of cell membranes, which leads to leakage of intracellular components and cell death [30]. According to Hua et al. [31], eugenol causes morphological alterations which occurred in the hyphae and conidiophores of *A. ochraceus* at 250 μg/ml using scanning electron microscopy. As well, eugenol increased ergosterol production by about 45–85%. Furthermore, eugenol has been revealed to inhibit the H + ATPase system, leading to intracellular acidification and cell death [32,33]. In addition, menthol and eugenol can act directly on the plasma membrane [[34], [35], [36]].

Antifungal activity of menthol and eugenol was also assessed by detecting MIC and MFC using the liquid dilution method. With this method, better conditions for the contact of menthol and eugenol with fungal spores and for their homogeneous diffuse into the medium are assured, as reported by Prakash et al. [16].The MIC values for complete inhibition of growth of *A. parasiticus* were recorded at 400 μg/ml and 300 μg/ml, respectively. Hua et al. [31] revealed that the MIC of eugenol against *A. ochraceus* was 600 μg/ml and Ju et al. [30] reported that the MIC of eugenol against *A. niger* was 250 μg/ml, which indicates that different species may possess different sensitivity towards the same antifungal. Both components were fungicidal in nature and the MFCs against *A. parasiticus* were higher than the MICs of menthol (>400 μg/ml) and eugenol (300 μg/ml).With such a low MIC value, menthol and eugenol, would require lower doses to inhibit mold infestation in food, allowing them to be formulated as ideal plant-based antimicrobials.

**Fig. 3:**
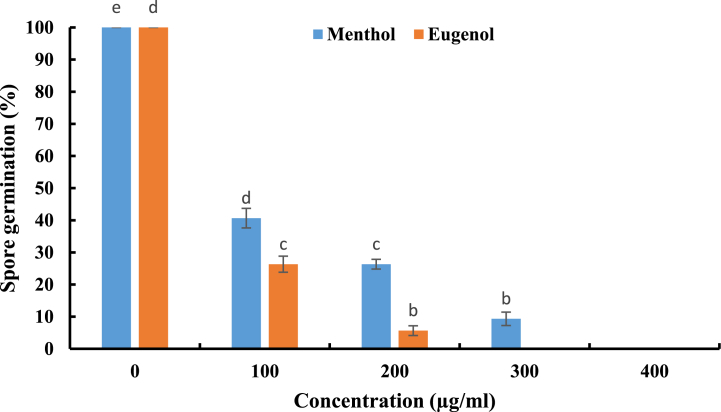
Effect of menthol and eugenol on spore germination of *A. parasiticus* (CBS 100926^T^). Values are means (n = 3) ± SD.

Spore is an important structure for the propagation of fungi. Based on the obtained results, menthol and eugenol showed inhibition of spore germination of A. parasiticus (CBS 100926T) at lower doses tested (Fig. 3). Significant efficacy of menthol and eugenol (p < 0.05) on the spore germination of *A. parasiticus* (CBS 100926T) was noted, after statistical analysis of the obtained results. Menthol (90.67%), eugenol (94.34%) had highest inhibitory effect on A. parasiticus (CBS 100926T) at 300 μg/ml and 200 μg/ml, respectively. Moreover, both menthol and eugenol exhibited a complete (100%) inhibition at 400 μg/ml.

The activity of several constituents on spore germination were also investigated. The activity of several constituents on spore germination were also investigated. Fernenol, arundoin, and a combination of stigmasterol and -sitosterol strongly inhibited *Colletotrichum gloeosporioides* spore germination and subsequent germ tube with 45.8, 62.3, and 86.9 mg/l, respectively [37]. Nishino et al. [38] showed that 1-Phenyl-3-pentanone from an edible mushroom had significantly inhibitory spore germination of some plant-pathogenic fungi 35 ppm. Tian et al. [39] showed that nerol reduced spore germination of *A. flavus* at 0.8 μl/ml. Thymol has been depicted to inhibit spore germination of fungi such as *Rhizoctonia solani*, *Alternaria mali* and *Phytophthora capsici* [40]. Wang et al. [41] showed that spore germination *of A. niger* with an increasing concentration of nerol. More recently, Zhou et al. [36] reported that carvacrol and eugenol inhibited *R. stolonifer* germination.

The efficacy of menthol and eugenol on spores may be as a consequence of enzymes denaturation implicated in spore germination or interference with amino acids involved in germination [42,43].

Menthol and eugenol had great antifungal activity when applied to coffee beans those are artificially contaminated with *A. parasiticus*. Green coffee beans were fumigated with both compounds as potential natural food preservatives. Menthol and eugenol showed remarkable efficacy in fumigated green coffee beans sample during storage up until 12 months supplying protection from *A. parasiticus* contamination of 62.5% for menthol and 73.21% for eugenol as presented in Table 1.

**Table 1:** Control of *A. parasiticus* (CBS 100926^T^) of green coffee beans after storage fumigated with menthol and eugenol.

Sample	Concentration (μg/ml)	*A. parasiticus* isolates		% protection
		Control	fumigation	
Menthol	400	112	42	62.50
Eugenol	300	112	30	73.21

*Aspergillus* species reduce food quality and safety by the production of spoilage enzymes that induce discoloration and abnormal flavour, in addition to the synthesis of mycotoxins such as aflatoxins. The most important step of protecting the coffee beans from fungal and mycotoxin contamination is their proper storage after processing [44]. Improving the quality (not only the final product but also during the storage and transportation) and streamlining of coffee processing, is critical to mitigating the negative effects of fungi and mycotoxins contamination. One solution is to use plant-based products, such as EOs and their major constituents.

Natural antifungals are eco-friendly and safer than synthetic chemical fungicides. The preservative effects in food items requires higher concentrations of EOs and their major compounds [14,45,46], which can be explained by the formation of food lipids an envelope around microorganisms to protect them antimicrobial agents [47]. As previously reported, greater concentrations of EOs are needs in foodstuffs than in laboratory media, possibly because the lower water content of coffee beans compared to culture media can hinder the transport of antimicrobial molecules to the active site in the microbial cell [35]. According to the study of Hlebová et al. [48], the use of EOs appears to be an effective method for protecting stored coffee from harmful fungi without compromising the organoleptic properties (aroma and taste) of coffee beverages.

Although previous research has shown that the use of vapors is an appropriate method technique for controlling food contamination because it leaves no remaining components [46], their high volatility is the critical issue regarding their applications and shelf life. The microencapsulation method is a suitable technique to solve the problem. Encapsulation provide effective protection of natural compounds against chemical reactions and undesirable interactions with other food components. In addition, it improves solubility, decreases migration and preserves the stability of bioactive compounds during food processing and storage [[49], [50], [51], [52]].

## 4 Conclusion

The use of natural sources to control the contamination in food commodities and prolong their shelf life have received a raised attention, recently. Biologically active natural components possess the possibility to take the replace of synthetic fungicides. The EOs are also among the plant extracts alternatives that are applicable against the fungal contamination, thereby prolonging shelf life. Although most of the EOs have been presented to provent postharvest fungi under *in vitro* conditions, the studies on the *in vivo* effectiveness and practical activity of most of EOs have been very limited. The present study revealed menthol and eugenol can be effectively used to reduce fungal contamination caused by *A. parasiticus* during long-term storage. Furthermore, menthol and eugenol may be commercialized for the treatment of postharvest diseases, and possibly for other agricultural products.

## Funding

This research was supported by project PRFU/ALG-MICO (MESRS Grant No: D00L01UN150120180002)- Algeria. Funding for open access charge: Universidade de Vigo/CISUG.

## Author contribution statement

Yamina Ben Miri: Conceived and designed the experiments; Performed the experiments; Analyzed and interpreted the data; Wrote the paper. Ahmed Nouasri: Performed the experiments. Amina Benabdallah, Abderrahim Benslama, Zeynep Tacer-Caba: Analyzed and interpreted the data; Wrote the paper. Affaf Laassami, Djamel Djenane: Contributed reagents, materials, analysis tools or data. Jesus Simal-Gandara: Conceived and designed the experiments; Wrote the paper. Data availability statement: Data will be made available on request. Declaration of interest's statement: The authors declare that they have no known competing financial interests or personal relationships that could have appeared to influence the work reported in this paper.

## Declaration of competing interest

The authors declare that they have no known competing financial interests or personal relationships that could have appeared to influence the work reported in this paper.
